# Individual differences in relative fertility costs and fertility benefits and their effects on fertility desire for a second child in China: a latent profile analysis

**DOI:** 10.1186/s12978-019-0770-1

**Published:** 2019-07-18

**Authors:** Shi-Min Chen, Ying Zhang, Yi-Bao Wang

**Affiliations:** 1 Research Center of Jiangsu Public Security, China University of Mining and Technology, Xuzhou, 221116 China; 2 School of Public Administration, China University of Mining and Technology, Xuzhou, 221116 China; 3 School of Sociology and Psychology, Central University of Finance and Economics, Beijing, 100081 China

**Keywords:** Relative fertility costs, Fertility benefits, Fertility desire, Latent profile analysis, China

## Abstract

**Background:**

Fertility desire for a second child has been a lively topic since the implementation of the two-child policy in China. Chinese researchers have explored various factors influencing the fertility desire for a second child. However, there have not been studies on the individual differences in the relative fertility costs and fertility benefits and their effects on fertility desire for a second child.

**Methods:**

A total of 396 participants rated four kinds of relative fertility costs, four kinds of fertility benefits and their fertility desire for a second child. Latent profile analysis (LPA) was used to explore the individual differences in the relative fertility costs and fertility benefits and their effects on fertility desire for a second child.

**Results:**

Stepwise regression analysis showed that parenting joy, health risks, mutual care among siblings, the flourishing of family, and time pressure and opportunity cost significantly predicted the fertility desire for the second child. According to the latent profile analysis, the participants were classified into four classes. Participants in the lowest-cost/lowest-benefit and high-cost/medium-benefit classes had low fertility desire for a second child, while those in the low-cost/high-benefit and highest-cost/highest-benefit classes had high fertility desire.

**Conclusion:**

Fertility benefits have a stronger effect on the fertility desire for a second child than relative fertility costs. Fertility benefits should be paid more attention to than relative fertility costs.

## Plain English summary

Fertility desire for a second child has been a hot topic since the implementation of the two-child policy in China. All kinds of factors affecting the fertility desire for a second child have been explored. However, no studies have been conducted on the individual differences in the relative fertility costs and fertility benefits and their effects on fertility desire for a second child. A total of 396 participants scored four kinds of relative fertility costs, four kinds of fertility benefits and their fertility desire for a second child. Stepwise regression analysis showed that parenting joy, health risks, mutual care among siblings, the flourishing of family, and time pressure and opportunity cost significantly predicted the fertility desire for the second child. According to the latent profile analysis, the participants were classified into four classes. Participants in the lowest-cost/lowest-benefit and high-cost/medium-benefit classes had low fertility desire for a second child, while those in the low-cost/high-benefit and highest-cost/highest-benefit classes had high fertility desire. The results showed that fertility benefits have a more important influence on the fertility desire for a second child than relative fertility costs. It is suggested that the government should implement favourable policies to decrease women’s time pressure and opportunity cost. We foresee that the fertility benefits that might gradually become apparent over time would encourage more people to bear one more children.

## Introduction

The one-child policy was first implemented in 1980 to control the population size in China. However, given the ageing society, the one-child policy was phased out, and the two-child policy has been gradually implemented. Couples in which both the husband and wife were single children were allowed to bear two children as of November 2011. Couples in which one partner was a single child were permitted to bear two children as of December 2013. As of October 2015, all couples are allowed to bear two children. Fertility desire is an important predictor of fertility behaviour. When people want to give birth to a child, they take actions to become pregnant; otherwise, they take contraceptive actions. Therefore, fertility desire and its influencing factors have become a significant issue that both the public and government are closely concerned about since the implementation of two-child policy in China. Fertility desire for a second child has been a heated topic of conversation when people get together. A great many studies have explored the fertility desire for a second child in China and its influencing factors.

### Fertility desire for a second child in China

Researchers have investigated fertility desire for a second child in China. The Chinese General Social Survey (*N* = 7810 participants from 29 Chinese provinces) carried by Survey and Data Center of China from Renmin University of China indicated that 68.8% Chinese residents wanted to give birth to their second child [1]. The Chinese Social Survey launched by the Institute of Sociology, Chinese Academy of Social Sciences, in 25 provinces in China (*N* = 4194) showed that 81.7% of rural residents wanted to bear their second child [2]. As for cities, 65% of residents in Qingdao, Shandong Province wanted to bear a second child [3], whereas this figure was only 41.4% in Tianjin [4] and 34.8% in Shanghai [5]. In total, the fertility desire for a second child in rural China is higher than that in cities and the bigger the city is, the lower the fertility desire is.

According to annual Statistical Bulletin on National Economic and Social Development issued by National Bureau of Statistics of the People’s Republic of China, the number of newborns in China was 16.87 million in 2015 [6], 17.86 million in 2016 [7], 17.23 million in 2017 [8] and 15.23 million in 2018 [9]. It can be seen that the number of births of newborns began to decrease in 2017, and declined sharply in 2018, which is beyond many people’s expectations. Both the government and researchers hope to further explore the desire for a second child in order to enhance fertility rate.

### Relative fertility costs and fertility benefits

Chinese researchers have explored various factors influencing the fertility desire for a second child. These factors include women’s age [4, 10], women’s education [4], the organizational type of women’s job (within or outside the political system) [11], family income [12, 13], educational expenditure and housing prices [1], family structure and intergenerational care [12, 14, 15], preference for a boy [16, 17], parental fertility expectation for grandchildren [14], family size of patrilineal origin [14].

However, there are few studies exploring the effect of fertility costs and benefits on fertility desire from a comprehensive perspective. The reason for having a child not only is based on biological predisposition and social coercion but also is a rational choice by couples [18]. The rational choice theory of fertility considers that couples are rational economic persons who take the costs and benefits of fertility into account and make the best use of limited family resources to fulfil the maximum utility for the family. We would like to explore the influencing factors of the fertility desire for a second child on the basis of fertility costs and benefits in this study. Furthermore, we coin the term “relative fertility cost” to better measure fertility cost. Relative fertility cost is equal to the ratio of the absolute fertility costs to fertility resources, which considers the influence of fertility resources and can better explain the fertility desire than the absolute cost. For example, there is a U-shaped relationship between the couple’s income and the fertility desire for a second child in China [12], which can be explained by the relative economic cost, i.e., the ratio of parental cost divided by family income. Assuming that the annual incomes of low-income, middle-income and high-income families are ¥50,000, ¥200,000 and ¥1 million, respectively, and their parental costs are ¥10,000, ¥50,000 and ¥100,000 respectively, the relative economic costs of low-income, middle-income and high-income families are 0.2, 0.4 and 0.1, respectively. The relative economic cost of low-income and high-income families is lower than that of the middle-income family. Another examples is that potential (or actual) childcare provision from grandparents promotes fertility desire [14], which decreases the cost of time and opportunity. Therefore, we use the term “economic pressure” instead of “economic cost”, and “time pressure and opportunity cost” instead of “time pressure and opportunity cost” in this study.

Relative fertility costs include economic pressure, time pressure and opportunity costs, conflict among siblings, health risks and so on. Different relative fertility costs affect fertility desire in different ways. First, economic pressure has been a key factor that has seriously inhibited the fertility desire of the public because the parenting costs, which consists of the cost of education and housing purchases, are soaring [17, 19]. Second, the more education females acquire, the higher the opportunity costs will become. More education increases women’s economic opportunity cost of leaving the labour market to care for children and their desire for personal fulfilment [20–24]. Therefore, increase in the wife’s schooling decreases family size. Third, when parents give birth to a second child, they are likely to reduce the care to the first child to some extent, which causes some older children to object their parents bearing a second child. Furthermore, it is inevitable that there is conflict between the first child and the second child. Both may interfere with parents’ fertility desire for a second child. In addition, the fertility desire of women decreases after they are more than 35 years old, especially 40 years old because childbearing often has higher risk of adverse pregnancy outcomes. Women at advanced maternal age have a higher risk of chromosomal abnormalities, miscarriage, and birth before 34 \s of gestation than younger women [25].

Many fertility benefits have been introduced in previous studies such as parenting joy, mutual care among siblings, elderly care, and the flourishing of family. Different fertility benefits influence fertility desire in different ways. First, Preference Theory and other ideology-based theories stressed the importance of values for fertility desire [26]. Of the three groups of women (work-focused, home-focused, or combined focus), family-focused women who enjoy their family life, keep intimate contact with family members and regard the growth of children as their most important achievement are more willing to bear more children [26, 27]. Moreover, fertility desire is influenced by the life course of the family [28]. Those who grow up with siblings, get along well with them and get help from them are inclined to bear more children [4, 17]. In addition, family support for the elderly is still the most important pattern of elderly care in China, though social security has developed rapidly and the idea of raising sons to support the elderly has been weakening. According to 2010 census data, there were approximately 1 million families who lost their single child [29], which was a devastating blow to those families. Many people tend to bear one more child to prevent the risk of losing the single child. Finally, the flourishing of family is one of the most important dreams of Chinese. The rich hope to have more children to take over the family business, and the poor wish to have more children to help the family to get out of the difficult life situation [4].

### Variable-centered approach and person-centered approach

When we explore the effect of multiple predictors on dependent variable in a population, there are two approaches: variable-centered approach and person-centered approach [30]. The variable-centered approach assumes that all individuals from a sample are drawn from a single population for which a single set of “averaged” parameters can be estimated; whereas the person-centered approach consider the possibility that the sample might include multiple subpopulation characterized by different sets of parameters. The variable-centered approach detects general associations that summarize an entire population, while the person-centered approach classifies similar individuals into unique subpopulations that may be based on very complex patterns of many variables, and then understand the relationship of these subpopulations with predictors, correlates, or outcomes. The results of the person-centered approach provide more specificity than variable-centered approach because multiple subpopulations are described separately the entire sample together.

### Aim of this study

There are two limitations in previous studies. One is that previous researches only explored various factors have influence on fertility desire for a second child. However, what effect size of these factors having effect on fertility desire has not been probed into. We would like to inquire into this problem with stepwise regression.

The other is that previous studies only investigated the influence of different factors on fertility desire with variable-centered approach, rather than person-centered approach. The fertility desire for an addition child is influenced by many factors and the individual difference is great. It is believed that is it useful to explore the individual difference of relative fertility costs and fertility benefits and their effect on fertility desire with person-centered approach. We would like to explore this problem with latent profile analysis (LPA). LPA is a multivariate approach which identifies groups based on individual’s observed response patterns to categorize the participants into optimal groups [31]. Besides, this method is based on probability, which can take uncertainty of individual’s class membership into account [32].

## Methods

### Data collection procedure

The data collection procedure included the following steps. First, we obtained ethical approval from the Research Ethics Committee of the host university. Second, the questionnaire was uploaded to the website of “Questionnaire Star”, which was the most widely used questionnaire website in China. Third, the electronic questionnaire forwarded by the social software WeChat and QQ. The principles of voluntariness and confidentiality were underlined in the instruction section of the questionnaire. Participants were reminded that their participation was voluntary and that they could discontinue it at any time. They were assured that their responses would be kept confidential. They were also asked if they would like their test results to be used as data sources for this study and published. The survey was conducted from October 2017 to December 2017.

### Participants

It was required that the participants be between 18 and 45 years old and not have two children. A total of 396 valid questionnaires were obtained from 52 administrative cities in China (from 209 male and 187 female respondents). The mean age of the sample was 31.4 years (SD = 7.5), and it comprised 4 primary school graduates, 162 middle school graduates, 177 college graduates, 48 master’s degree graduates and 5 doctoral degree graduates. A total of 305 were junior employees, 82 were mid-level employees, and 9 were senior employees. Two hundred and forty-four participants lived in urban districts, 61 in counties, 66 in towns and 25 in rural areas. One hundred and five people rented apartments, 203 owned one flat, 66 owned two, 8 owned three or more, and 14 persons did not indicate their living condition. One hundred and fifty-seven were single children, and 239 were not. One hundred and ninety-one had no children, and 205 had one child.

### Measurement

The three kinds of relative fertility costs of economic pressure, time pressure and opportunity cost, conflict among siblings were measured by the 15-item Fertility Costs Subscale in the Fertility Cost-Utility Questionnaire [33]. Example items include as follows: “The high cost of child rearing puts great pressure on the family economy”, “Childbearing has a negative impact on career development”, “I worry about the conflict between the first child and the second child”. The health risks were assessed by three items (e.g., “Childbearing has negative effects on women’s health, and I worry my (my partner’s) health will become poor if I (my partner) bear one more child”). Responses are given on a 5-point scale ranging from 1 (strongly disagree) to 5 (strongly agree) to describe the reason that they want or don’t want to have a second child. The reliability of the Fertility Cost Subscale in this study was Cronbach’s *α =* 0.834.

The four kinds of fertility benefits (the parenting joy, mutual care among siblings, elderly care, and the flourishing of family) were measured by the 16-item Fertility Utility Subscale in the Fertility Cost-Utility Questionnaire [33]. Example items include as follows: “Children bring a lot of happiness to me and my family members as they grow up”, “Multiple children can help and promote each other”, “Multiple children can provide parents with more elderly care”, “More children is beneficial to the flourishing of family”. Responses are given on a 5-point scale ranging from 1 (strongly disagree) to 5 (strongly agree) to describe the reason that they want or don’t want to have a second child. The reliability of the Fertility Benefit Subscale in this study was Cronbach’s *α* = 0.872.

The fertility desire for a second child was assessed by one question, which asked participants to indicate their fertility desire for a second child on a 5-point scale ranging from 1 (strongly reluctant) to 5 (strongly voluntary).

### Data analysis

LPA was conducted with Mplus 7.0 software. Four criteria were used for deciding the best fitting model. The first criterion was the adjusted Bayesian information criterion (aBIC) which is the most reliable indicators of true model fit [34]. Additionally, we used Lo-Mendell-Rubin likelihood ratio test (LMR) [35] and bootstrap likelihood ratio test (BLRT). These two indicators assess the model fit between two nested models. For example, a nonsignificant LMR *p* value for a three-class model indicates that the two-class model fits better than the three-class model. Finally, entropy value was also used to evaluate classification accuracy. Values closer to 1.0 indicate better classification accuracy. While, values greater than 0.80 can be considered to have adequate classification accuracy [36].

In addition, the effect size of the mean difference test was measured with Cohen’s d. The effect size was small when 0.2 ≤ d < 0.5, medium when 0.5 ≤ d < 0.8 and large when d > 0.8 [37, 38].

## Results

### Descriptive statistics and correlations

The means, standard deviations, and correlations among relative fertility costs, fertility benefits and fertility desire for a second child are shown in Table 1. As observed, the fertility desire for a second child was at a medium level (M = 2.97, SD = 1.09). The fertility desire for a second child had a significant negative correlation with health risks, economic pressure, time pressure and opportunity cost, and conflict among siblings and a significant positive correlation with parenting joy, mutual care among siblings, elderly care, and the flourishing of family.

**Table 1:** Means, standard deviation, and bivariate correlation of relative fertility costs, fertility benefits and desire

	1	2	3	4		5	6	7	8	9
1.Eco_P	1									
2. Ti&op_C	.637**	1								
3. Con_S	.238**	.403**	1							
4.Hea_R	.374**	.559**	.442**	1						
5. Par_J	0.024	−0.064	−.156**	−0.049	1					
6.Mu_C	.129*	0.092	−0.023	0.098	.505**	1				
7.Eld_C	−0.069	−.158**	0.052	−.101*	.318**	.498**	1			
8. Flou_F	−.188**	−.122*	.128*	−0.025	.237**	.320**	.603**	1		
9.Fer_Int	−.145**	−.212**	−.106*	−.218**	.292**	.283**	.295**	.264**	1	
M	3.41	3.31	2.89	3.18	3.68	3.45	3.22	2.95	2.97	
SD	0.85	0.83	1	0.74		0.74	0.73	0.78	0.79	1.09

Note: *n* = 396, **p < 0.01, ***p < 0.001, *Eco_P* economic pressure, *Ti&op_C* time pressure and opportunity cost, *Con_S* conflict among siblings, *Hea_R* health risks, *Par_J* parenting joy, *Mu_C* mutual care among siblings, *Eld_C* elderly care, *Flou_F* the flourishing of family, *Fer_Int* fertility desire

### Prediction of relative fertility costs and fertility benefits on fertility desire

Stepwise regression analysis was performed to analyse the prediction of the four kinds of relative fertility costs and the four sorts of fertility benefits on fertility desire for a second child. Multicollinearity diagnosis was conducted. The least tolerance was 0.667 (bigger than 0.1) and the maximum VIF was 1.500 (less than 10), which indicated that there was not serious multicollinearity problem. It could be seen from Table 2 that two kinds of fertility cost (health risks, time pressure and opportunity cost) and three sorts of fertility benefit (parenting joy, mutual care among siblings, the flourishing of family) significantly predicted the fertility desire for a second child. Parenting joy accounted for the most variance of the fertility desire for a second child with the determination coefficients (△*R*^2^) of 8.5% among the five predictors. The health risks, mutual care among siblings, the flourishing of family accounted for 4.2, 3.9, 2.1% of the variance of the fertility desire, respectively. Time pressure and opportunity cost accounted for the least variance of the fertility desire with the determination coefficients (△*R*^2^) of 0.8%. Totally, the fertility benefit accounted for 14.5% variance of the fertility desire for a second child, whereas the fertility cost accounted for 5.0% variance of the fertility desire.

**Table 2:** Stepwise regression of relative fertility cost and fertility benefit on fertility desire

Predictor	B	SE	β	t	F	ΔR^2^
Constant	1.843	0.385		4.790***	18.872***	
Par_J	0.216	0.078	0.147	2.765**		0.085
Hea_R	−0.239	0.081	−0.164	−2.972**		0.042
Mu_C	0.277	0.082	0.186	3.366**		0.039
Flou_F	0.209	0.067	0.152	3.121**		0.021
Ti&op_C	−0.144	0.073	−0.11	−1.978*		0.008

Note: *n* = 396, **p < 0.01, ***p < 0.001, *Par_J* parenting joy, *Hea_R* health risks, *Mu_C* mutual care among siblings, *Flou_F* the flourishing of family, *Ti&op_C* time pressure and opportunity cost

### Latent profile analysis of relative fertility costs and fertility benefits

In order to determine the optimal number of classes, LPA was conducted. Specifically, we included eight indicators in this analysis: economic pressure, time pressure and opportunity cost, conflict among siblings, health risks, parenting joy, mutual care among siblings, elderly care, and the flourishing of family. The fit indices of models withtwo to five class solutions are summarized in Table 3. The four-class solution has been found to show the optimal fit for the data. The aBIC values of the four-class solution were less than those of the two-class solution and the three-class solution. The LMR *p* value for the four-class solution was significant, whereas the LMR *p* value for the five-class solution were not significant. These indicate that the four-class model was significantly better than the three-class model but not significantly better than the five-class model. Finally, the entropy value was larger than 0.80.

**Table 3:** Latent class fit indices of relative fertility cost and fertility benefit

Model	aBIC	LMR(*p*)	BLRT (*p*)	entropy
2-class	7370.226	0.002	0.000	0.698
3-class	7252.556	0.040	0.000	0.717
4-class	7131.132	0.020	0.000	0.801
5-class	7043.365	0.553	0.000	0.800

**Fig. 1:**
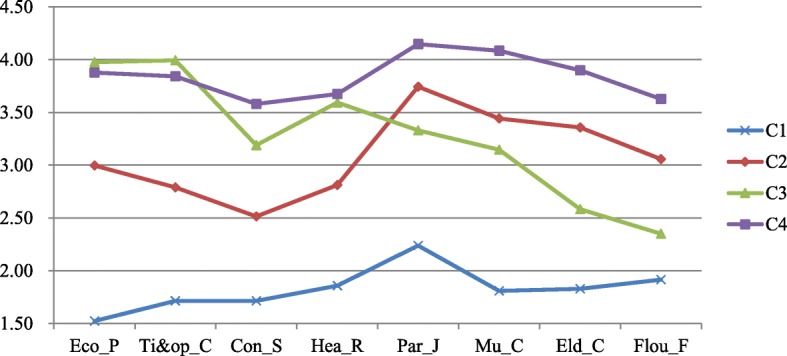
Latent profile of relative fertility costs and fertility benefits. Note: Eco_P = economic pressure, Ti&op_C = time pressure and opportunity cost, Con_S = conflict among siblings, Hea_R = health risks, Up_J = upbringing joy, Mu_C = mutual care among siblings, Eld_C = elderly care, Flou_F = the flourishing of family

The conditional means, standard deviations (SD), and latent class probabilities for the four-class model are showed in Table 4. The latent profiles in the four class model are depicted in Fig. 1. Class 1 comprised 1.8% (*n* = 7) participants of the sample. The conditional means of the four kinds of relative fertility costs and four kinds of fertility benefits were between 1.5–2.3. The values in this profile were the lowest among the four classes; therefore, we label this class as lowest-cost/lowest-benefit. Besides, Class 2 comprised 51.5% (*n* = 204) participants in this sample. The conditional means of the four kinds of relative fertility costs were between 2.5–3.0, and those of the four kinds of fertility benefits were between 3.0–3.8; therefore, this classwas named the low-cost/high-benefit. Class 3 comprised 28.0% (*n* = 111) participants in this sample. The conditional means of the four kinds of relative fertility costs were between 3.1–4.0, and those of the four kinds of fertility benefits were between 2.0–3.5; therefore, this class was labeled as the high-cost/medium-benefit class. Class 4 comprised 18.7% (*n* = 74) of the sample. The conditional means of the four kinds of relative fertility costs and four kinds of fertility benefits were all above 3.5. The values in this class were the highest among the four classes; therefore, this class was labeled as the highest-cost/highest-benefit.

**Table 4:** Conditional mean, SD and LCP of 4-class model

Indicator	C1		C2		C3		C4	
	M	SD	M	SD	M	SD	M	SD
Eco_P	1.52	0.77	3.00	0.67	3.98	0.68	3.88	0.66
Ti&op_C	1.71	0.45	2.79	0.6	3.99	0.54	3.84	0.54
Con_S	1.71	0.76	2.51	0.8	3.19	1.03	3.58	0.88
Hea_R	1.86	0.63	2.81	0.6	3.59	0.56	3.68	0.67
Par_J	2.24	0.85	3.74	0.67	3.33	0.72	4.15	0.54
Mu_C	1.81	0.69	3.44	0.63	3.15	0.66	4.09	0.54
Eld_C	1.83	0.67	3.36	0.6	2.58	0.63	3.90	0.55
Flou_F	1.91	0.76	3.06	0.68	2.35	0.61	3.63	0.61
LCP	0.018		0.515		0.28		0.187	

Note: *Eco_P* economic pressure, *Ti&op_C* time pressure and opportunity cost, *Con_S* conflict among siblings, *Hea_R* health risks, *Par_J* parenting joy, *Mu_C* mutual care among siblings, *Eld_C* elderly care, *Flou_F* the flourishing of family, *LCP* latent class probability

### Fertility desire of different latent classes

Table 5 shows the descriptive statistics of the fertility desire of different latent classes. As observed, there is little difference in the fertility desire for a second child between the lowest-cost/lowest-benefit and high-cost/medium-benefit classes. The mean difference test showed that there was no significant difference between the two classes (*t* = − 0.154, *p* > 0.05). There was also little difference in the fertility desire between the low-cost/high-benefit and highest-cost/highest-benefit classes. The mean difference test showed that there was no significant difference between the two classes (*t* = 0.770, *p* > 0.05). In other words, there were two levels of fertility desire. The fertility desire for a second child was relatively low for lowest-cost/lowest-benefit and high-cost/medium-benefit classes (merged as the low-desire class), whereas the fertility desire for a second child was relatively high for low-cost/high-benefit and highest-cost/highest-benefit classes (merged as the high-desire class). According to the latent class probability in Table 4, the low-desire class accounted for only 29.8% of the participants, whereas the high-desire class accounted for 70.2%.

**Table 5:** Descriptive statistics of the fertility desire of different latent classes

Class	N	M	SD
lowest-cost/lowest-benefit	7	2.29	1.89
low-cost/high-benefit	204	3.19	0.99
high-cost/medium-benefit	111	2.40	0.98
highest-cost/highest-benefit	74	3.30	1.08

The difference of fertility desire between low-desire class and high-desire class is presented in Table 6. The mean difference test indicated that the fertility desire between the two classes was significantly different with large effect size of Cohen’s d 0.81(> 0.80).

**Table 6:** Difference of fertility desire for a second child between low-desire class and high-desire class

Variable	Low-desire class			high-desire class			t	d
	M	SD	n	M	SD	n		
Fer_Int	2.39	1.04	118	3.22	1.02	278	−7.39***	0.81

Note: ***p < 0.001, *Fer_Int* fertility desire

### Discussion

### Effect of relative fertility costs and fertility benefits on fertility desire

The first purpose of this study was to investigate the effect size of different relative fertility costs and fertility benefits on the fertility desire for a second child. The results of stepwise regression analysis showed that three kinds of fertility benefits significantly have influence on fertility desire. Parenting joy accounted for the most variance in the desire to bear an additional child. The possible reason is that most Chinese women are still family oriented, and their most important values are still to help their husbands and teach their children. Additionally, with the development of society, many Chinese men have begun to pay more attention to fostering their children, and they view the growth of their children as their accomplishment and as a source of well-being. Mutual care among siblings is the second fertility benefit influencing the childbearing desire. The one-child policy was implemented for more than 30 years. Social relationships are indifferent in cities. Many single children often felt lonely because they had no siblings and kept limited contact with other partners. Therefore, having grown up, they hope that their children can have one sibling to accompany them. The flourishing of family is the third fertility benefit having an effect on the desire to have a more child. Blood relationships are the most important relationship in China. Having a thriving family and plenty of children and grandchildren are the wishes of a great deal of Chinese. However, elderly care did not significantly predict the fertility desire for a second child, which could be explained in two ways. The importance of family support for the elderly has decreased with the development of social security. In addition, with the development of Chinese society, a large number of children leave their parents when they grow up, which causes many parents to reduce their expectation that their children will take care of them when they are old.

Two kinds of relative fertility costs have significant effects on fertility desire. Health risks accounted for the most variance in the fertility desire for a second child, which could be explained in two ways. Many women over 35 years old have missed the best age for childbirth because of the one-child policy that was carried out for more than 30 years. Furthermore, more and more women get married at over 28 years old, and many of them worry about the adverse pregnancy outcome to bear their second child. The other fertility cost that has an impact on the childbearing desire is time pressure and opportunity cost. With the development of Chinese society, more and more Chinese women receive a higher education and enter the work force. They worry that giving birth to their second child would have negative impact on their career. Counter to our expectation, economic pressure did not significantly predict fertility desire. As the Chinese proverb says, “If you are rich, you raise your children in a prosperous way; however, if you are poor, you foster your children in an economical way.” Those parents who want to give birth to their second child will keep expenditures within the limits of income if they are not wealthy. However, those couples who do not want to bear their second child often use economic pressure as their excuse. Finally, conflict among siblings did not have a significant effect on the desire to have an additional child. The possible reason is that this problem is not the important influencing factor of fertility desire, though some parents might worry about the objection of the older child.

The second purpose of this study is to explore the individual differences in relative fertility costs and benefits and their influence on fertility desire for a second child. The participants were classified into four classes by using latent profile analysis: lowest-cost/lowest-benefit, low-cost/high-benefit, high-cost/medium-benefit and highest-cost/highest-benefit. There were two levels of fertility desire for the four classes. Though the relative fertility costs of the lowest-cost/lowest-benefit and high-cost/medium-benefit classes were different, their fertility benefits were relatively low (shown in Fig. 1), and their fertility desire for a second child was also low. Similarly, though the relative fertility costs of the low-cost/high-benefit and highest-cost/highest-benefit classes were different, their fertility benefits were relatively high (shown in Fig. 1), and their fertility desire for a second child was also high. Thus, it can be observed that fertility benefits have a determinant effect on fertility desire, though relative fertility costs also have an important influence. Therefore, we should pay more attention to fertility benefits than relative fertility costs.

### Highlights of this study

This study yielded the following highlights. First, we combined relative fertility costs and fertility benefits to predict the fertility desire for a second child in China. Previous studies often predicted the fertility desire with few relative fertility costs or fertility benefits separately. We used four kinds of relative fertility costs and four kinds of fertility benefits comprehensively to predict the fertility desire. Moreover, we explored the individual differences in relative fertility costs and fertility benefits by using latent profile analysis, which has not been studied in previous studies. Additionally, this study showed that fertility benefits have more important effects on fertility desire than relative fertility costs, whereas previous studies paid more attention to the influence of relative fertility costs on fertility desire.

### Practical implication

This study is enlightening to help the government improve the fertility desire and fertility rate. First, the government should take action to reduce relative fertility costs. Older age causes high health risks for women. The government should guide women to make fertility plans to reduce health risks in the future under the two-children policy. Furthermore, more and more women receive a higher education and enter the work force, which greatly enhances time pressure and opportunity cost and decreases the fertility desire. The government should implement favourable policies to reduce time pressure and opportunity cost for women. The maternity leave changed from 98 + 30 days (extra 30 days for late childbearing) to 128 or 158 days in many Chinese provinces in 2017. However, such preferential policy is far from what is needed. Extended maternity leave should be considered. In addition, it is predicted that the fertility benefits including parenting joy, mutual care among siblings and the flourishing of family might gradually show up over time, which would encourage more people to bear one more child.

### Limitations and future directions

Despite its contributions, the study has several limitations that should be acknowledged. The first limitation is that the sample consisted of only 396 participants recruited on the basis of convenient sampling through the internet, which could not assure sample representativeness. Future research can enlarge the sample size on the basis of representative sampling. The second limitation is that we look into the fertility desire only of individuals and not couples, which could better predict fertility behaviour. Future research can investigate the fertility desire of couples to better predict fertility behaviour. Thirdly, the figures used to account for the relative economic cost in this study is assumed data on the basis of daily experience rather than real data from survey, which should be collected to enhance persuasion in the future. Finally, fertility costs and benefits are constantly changing. Future studies should keep inquiring into fertility costs and benefits and their influence on fertility desire and behaviour.

## Conclusions

Parenting joy, health risks, mutual care among siblings, the flourishing of family, and time pressure and opportunity cost significantly predicted the fertility desire for a second child. Participants could be classified into four classes according to relative fertility costs and benefits by using latent profile analysis: lowest-cost/lowest-benefit, low-cost/high-benefit, high-cost/medium-benefit and highest-cost/highest-benefit. Participants in the lowest-cost/lowest-benefit and high-cost/medium-benefit classes had low fertility desire for a second child, whereas those in the low-cost/high-benefit and highest-cost/highest-benefit classes had the high fertility desire. Fertility benefits have a stronger influence on fertility desire for a second child than relative fertility costs. It is suggested that the government should implement favourable policies to decrease women’s time pressure and opportunity cost. We predict that the fertility benefits that might gradually become apparent in future would encourage more people to have one more child.

## Data Availability

The data analyzed for this study are available from the corresponding author on request.

## Abbreviations

LCP: Latent class probability
LPA: Latent profile analysis
